# RCT: SPECIALIST NURSE-LED EXERCISE REHABILITATION IN AECOPD- IMPACT ON ENDURANCE AND QUALITY OF LIFE

**DOI:** 10.1093/geroni/igae098.2850

**Published:** 2024-12-31

**Authors:** Jiahui Ma, Hongjing Lin, Zeyu Qin, Zijing Chen

**Affiliations:** The First Hospital Of Jilin University, Changchun, Jilin, China (People’s Republic); The First Hospital Of Jilin University, Changchun, Jilin, China (People’s Republic); The First Hospital of Jilin University, Changchun, Jilin, China (People’s Republic); The First Hospital Of Jilin University, Changchun, Jilin, China (People’s Republic)

## Abstract

This prospective randomized controlled trial (RCT) evaluates the efficacy of a specialist nurse-led exercise rehabilitation program, grounded in the Patient Health Engagement (PHE) model, for patients with acute exacerbations of COPD (AECOPD). 84 patients with AECOPD were recruited from a Changchun, China, hospital and randomly assigned to intervention or control groups (42 in each). The 12-week intervention featured conventional exercise rehabilitation for the control group and a novel program for the intervention group, incorporating personalized exercise prescriptions led by specialist nurses. Baseline and 3-month post-discharge assessments included 6-minute walk distance (6-MWD) for exercise endurance and SF-36 for quality of life. Participants, with a mean age of 65.70 ± 6.13 years, exhibited severe airflow obstruction (FEV1: 39.48 ± 10.50%) and impaired motor ability (6-MWD: 282.10 ± 92.34m). After 12 weeks of intervention, the 6-MWD in the intervention group was 371.69 ± 65.09m, while the control group was 324.95 ± 74.96m. Both groups demonstrated improved exercise endurance (t1=37.899,95%CI,84.12-97,45,P1 < 0.001;t2=7.766,95%CI,31,60-66.79,P2 < 0.001), yet the intervention group displayed significantly greater enhancement in 6-MWD (90.80 ± 5.36, vs 49.20±14.16, t=-9.537,P < 0.001), indicating clinical significance. Furthermore, the intervention group exhibited higher quality of life scores than the control group, with statistical significance (t=2.988,P<0.05). This RCT underscores the specialist nurse-led exercise rehabilitation program’s effectiveness in enhancing exercise tolerance, improving compliance, and elevating the quality of life in patients with COPD, contributing valuable insights to rehabilitation nursing for patients with COPD.

